# Successful Treatment of Overlapping Myelin Oligodendrocyte Glycoprotein Antibody-Associated Disease and Glial Fibrillary Acidic Protein Astrocytopathy With Plasma Exchange

**DOI:** 10.14740/jmc5290

**Published:** 2026-03-27

**Authors:** Chisa Ashida, Tomohiro Suzuki, Jun Sawada, Ryo Yuzawa, Syuichi Tetsuka, Koji Hosoya, Tomoko Ogawa, Ritsuo Hashimoto

**Affiliations:** a Department of Neurology, International University of Health and Welfare Hospital, 537-3, Iguchi, Nasushiobara, Tochigi 329-2763, Japan; b Department of Nephrology, International University of Health and Welfare Hospital, 537-3, Iguchi, Nasushiobara, Tochigi 329-2763, Japan

**Keywords:** MOGAD, GFAP astrocytopathy, Overlapping syndrome, Plasma exchange

## Abstract

Myelin oligodendrocyte glycoprotein (MOG) antibody-associated disease (MOGAD) and glial fibrillary acidic protein (GFAP) astrocytopathy are distinct autoimmune inflammatory disorders of the central nervous system (CNS) that occasionally overlap. A 69-year-old man presented with acute gait disturbance, followed by progressive impairment of consciousness, myoclonus, cerebellar ataxia, and hyponatremia. Initial brain magnetic resonance imaging (MRI) revealed a high-intensity lesion in the left thalamus on diffusion-weighted imaging (DWI) and multiple white matter lesions on fluid-attenuated inversion recovery (FLAIR) imaging. While serum anti-MOG antibodies were positive, the clinical features, including progressive impairment of consciousness and hyponatremia, were atypical for MOGAD alone, leading to the identification of anti-GFAPα antibodies in the cerebrospinal fluid (CSF). High-dose corticosteroid therapy (three courses of intravenous methylprednisolone pulse) resulted in only partial clinical improvement. However, subsequent simple plasma exchange (PE) with fresh frozen plasma (FFP) led to marked neurological recovery. The mechanism of recovery is thought to involve the removal of both pathogenic antibodies and pro-inflammatory mediators. By day 82 of hospitalization, the patient was able to ambulate independently. This case suggests that in overlapping autoimmune syndromes, early escalation to PE should be considered when conventional steroid therapy is insufficient.

## Introduction

Myelin oligodendrocyte glycoprotein antibody–associated disease (MOGAD) and glial fibrillary acidic protein (GFAP) astrocytopathy are inflammatory central nervous system (CNS) disorders with overlapping clinical presentations, including encephalopathy, ataxia, and myelitis [1–3]. Although both conditions typically respond to corticosteroids [1–3], the coexistence of multiple autoimmune antibodies may contribute to more complex clinical presentations and heterogeneous treatment responses [3–5]. Distinguishing the predominant pathogenic antibody and identifying overlapping syndromes are essential for appropriate management [6].

Differential diagnosis of these conditions includes infectious meningoencephalitis, paraneoplastic neurological syndromes, and other autoimmune encephalitis such as anti-N-methyl-D-aspartate (NMDA) receptor encephalitis, as these conditions require fundamentally different diagnostic approaches and therapeutic considerations.

We report a case of overlapping MOGAD and GFAP astrocytopathy that was refractory to steroids but was successfully treated with simple plasma exchange (PE) with fresh frozen plasma (FFP).

## Case Report

A 69-year-old man with no remarkable past medical history presented with acute gait disturbance. He had no headache or meningeal signs. Six days prior to onset, he had experienced symptoms of an upper respiratory tract infection. Upon admission, laboratory tests revealed hyponatremia (Na 124 mmol/L). Neurological examination showed impaired consciousness (Glasgow Coma Scale: E3V4M4; Japan Coma Scale: I-3∼II-10), hyperreflexia in the lower extremities, and gait instability primarily attributable to truncal ataxia and involuntary movements. No apparent muscle weakness or sensory deficits were observed, indicating that the gait disturbance at onset was predominantly cerebellar in origin. He subsequently developed postural tremor, myoclonus in the upper limbs, progressive cerebellar ataxia, and urinary retention. On day 11, the patient developed respiratory failure and required endotracheal intubation and mechanical ventilation.

**Figure 1:**
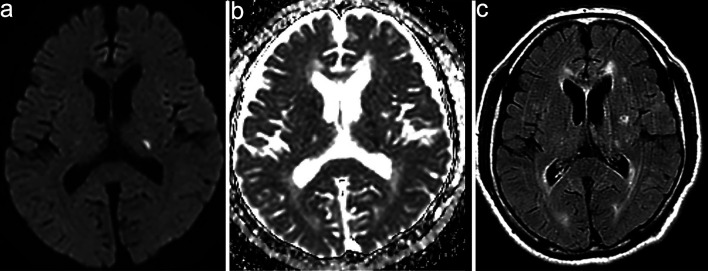
Magnetic resonance imaging (MRI) on admission. (a) High-intensity lesion in the left thalamus on diffusion-weighted imaging (DWI). (b) Corresponding low-intensity lesion on the apparent diffusion coefficient (ADC) map. (c) Fluid-attenuated inversion recovery (FLAIR) images demonstrating multiple spotty hyperintense white matter lesions.

Brain magnetic resonance imaging (MRI) on admission showed a high-intensity lesion in the left thalamus on diffusion-weighted imaging (DWI) with a corresponding low-intensity lesion on the apparent diffusion coefficient (ADC) map, along with multiple spotty white matter lesions on fluid-attenuated inversion recovery (FLAIR) (Fig. 1). Magnetic resonance angiography (MRA) showed no significant stenosis. Cerebrospinal fluid (CSF) analysis revealed pleocytosis (97/mm^3^), protein elevation (153 mg/dL), and positive oligoclonal bands (six bands). The immunoglobulin G (IgG) index was 0.959. Serum anti-MOG antibodies were positive (titer 1:16, Cell-based assay), whereas anti-aquaporin-4 (AQP4), anti-NMDA, and various paraneoplastic antibodies were negative (Table 1).

**Table 1:** Laboratory Findings

Category	Target antigen	Result
Autoimmune/demyelinating antibodies	MOG	Positive
	AQP4; NMDA; LGI1; CASPR2; APS; IgLON5	Negative
Paraneoplastic antibodies	Hu; Yo; Ri; CV2; PNMA2; AMPH; SOX1; Recoverin; Titin; Zic4; GAD65; Tr(DNER)	Negative
Systemic autoimmune antibodies	ANA; SSA; SSB; dsDNA; cardiolipin; CL-β2GP1; thyroglobulin; TPO	Negative
Infectious screening	HIV antigen/antibody	Negative

Antibodies other than anti-MOG, including those associated with paraneoplastic syndromes, were all negative at this time. AMPH: amphiphysin; ANA: antinuclear antibody; AQP4: aquaporin-4; APS: antiphospholipid syndrome; CASPR2: contactin-associated protein-like 2; CL-β2GP1: cardiolipin–β2 glycoprotein I; CV2 (CRMP5): collapsin response mediator protein 5; dsDNA: double-stranded DNA; GAD65: glutamic acid decarboxylase 65; HIV: human immunodeficiency virus; IgLON5: immunoglobulin-like cell adhesion molecule LON5; LGI1: leucine-rich glioma-inactivated 1; MOG: myelin oligodendrocyte glycoprotein; NMDA: N-methyl-D-aspartate; PNMA2 (Ma2): paraneoplastic Ma antigen 2; Recoverin: recoverin protein; Ri (ANNA-2): Ri antigen; SOX1: SRY-box transcription factor 1; SSA: Sjogren’s syndrome–related antigen A; SSB: Sjogren’s syndrome–related antigen B; Titin: titin protein; TPO: thyroid peroxidase; Tr(DNER): delta/notch-like epidermal growth factor–related receptor; Yo (PCA-1): Yo antigen; Zic4: zinc finger protein of the cerebellum 4.

**Figure 2:**
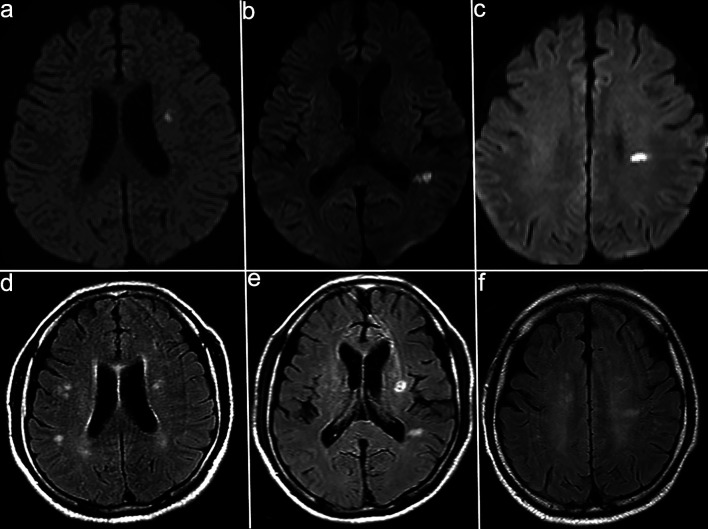
Serial diffusion-weighted imaging (DWI) showing the appearance of new lesions. (a–c) Follow-up magnetic resonance imaging (MRI) on day 7 (a), day 20 (b), and day 35 (c) shows small hyperintense lesions appearing sequentially in the left corona radiata and near the occipital left horn of the lateral ventricle. (d–f) Corresponding fluid-attenuated inversion recovery (FLAIR) images acquired at the same time points (a–d, b–e, and c–f pairs) demonstrate hyperintense white matter lesions.

**Figure 3:**
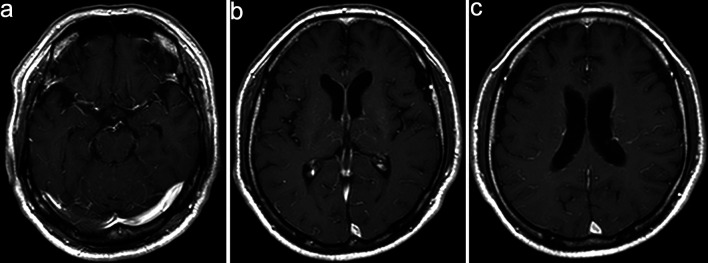
Contrast-enhanced magnetic resonance imaging (MRI) on day 11. (a–c) Contrast-enhanced MRI shows serpiginous perivascular enhancement in the basal ganglia along with leptomeningeal enhancement.

Although the positive results for anti-MOG antibodies led us to consider MOGAD as the initial diagnosis, the patient’s clinical course, including progressive impairment of consciousness and hyponatremia, was atypical. Serial follow-up MRI (DWI) revealed new small hyperintense lesions appearing sequentially in the left corona radiata and near the occipital horn of the left lateral ventricle (Fig. 2). On day 11, contrast-enhanced MRI showed serpiginous perivascular enhancement in the basal ganglia along with leptomeningeal enhancement (Fig. 3); however, these findings were not clearly consistent with the typical radial periventricular enhancement pattern reported in GFAP astrocytopathy. Given these atypical clinical and radiological findings, additional autoimmune antibody testing was performed, which confirmed the presence of anti-GFAPα antibodies in the CSF (Cell-based assay). Spinal cord imaging was performed and revealed no abnormalities. The patient had no visual symptoms, including visual disturbance, ocular pain, or visual field impairment. Dedicated optic nerve MRI was not obtained; however, the patient was evaluated by ophthalmology, which identified perivascular inflammation around the right optic nerve. Whole-body computed tomography (CT) screening and tumor markers revealed no malignancy.

The patient was treated with three courses of intravenous methylprednisolone pulse (IVMP) therapy (1,000 mg/day for 3 days per course). Following IVMP, serum sodium levels normalized and respiratory status improved, allowing successful weaning from mechanical ventilation. However, the response was partial, and impaired consciousness (Glasgow Coma Scale: E3V4M6; Japan Coma Scale: II-10), myoclonus, tremor, and severe cerebellar ataxia persisted. Simple PE with FFP (three sessions) was then initiated on days 40, 45, and 47, which resulted in significant neurological improvement, including restoration of clear consciousness (Glasgow Coma Scale: E4V5M6; Japan Coma Scale: 0). Myoclonus and postural tremor resolved, and cerebellar ataxia markedly improved, enabling the patient to sit and stand independently and eventually ambulate without assistance. However, urinary retention persisted and continued to require management. Following PE, the CSF cell count significantly improved from 235 to 10/mm^3^, and no new lesions were detected on brain MRI, and previously observed enhancement resolved; therefore, PE was discontinued after three sessions. He was discharged on day 82 with the ability to ambulate independently while continuing oral prednisolone (20 mg/day).

## Discussion

The coexistence of multiple autoantibodies, termed “overlapping syndrome,” has been increasingly reported [7]. Among these, the overlap of anti-MOG antibodies and anti-GFAP antibodies represents a unique clinical entity where two distinct pathogenic processes, demyelination and astrocytopathy, occur simultaneously.

In our patient, several clinical clues suggested that GFAP astrocytopathy was a major contributor despite the initial detection of MOG antibodies. First, the progressive encephalopathy, myoclonus, cerebellar ataxia, and urinary retention are highly characteristic of GFAP astrocytopathy [8]. While MOGAD can present with acute disseminated encephalomyelitis (ADEM)-like features [2], the severity of the consciousness impairment was more suggestive of an astrocytopathic process [9]. Second, the marked hyponatremia (Na 124 mmol/L) is a well-documented feature of GFAP astrocytopathy [8], likely due to the involvement of the hypothalamus, whereas it is uncommon in isolated MOGAD. Although the patient fulfilled the 2023 international diagnostic criteria for MOGAD [2], several clinical red flags including poor responsiveness to corticosteroid therapy and oligoclonal band positivity, indicated an atypical presentation for MOGAD, supporting GFAP astrocytopathy as the predominant pathogenic process.

Although both MOGAD and GFAP astrocytopathy are generally steroid-responsive and previously reported cases of overlapping MOGAD and GFAP astrocytopathy have shown favorable outcomes [3, 4], our patient exhibited a severe clinical course requiring multimodal immunotherapy. In the present case, high-dose corticosteroid therapy resulted in partial clinical improvement, including normalization of hyponatremia and recovery of respiratory function, allowing discontinuation of mechanical ventilation. However, impaired consciousness, myoclonus, tremor, and cerebellar ataxia persisted despite steroid therapy, indicating incomplete treatment response. Notably, most reported overlap cases recovered well after immunotherapy, whereas the present case required mechanical ventilation and escalation to PE. One possible explanation is advanced age, as older patients may be more susceptible to severe disease progression and poorer treatment responsiveness. Therefore, age and rapid clinical deterioration may represent important factors prompting early therapeutic escalation in overlapping autoimmune CNS syndromes.

The pathophysiology of this overlapping syndrome remains to be fully elucidated. However, it may involve “epitope spreading,” a phenomenon where tissue damage caused by the primary autoimmune attack releases secondary antigens, triggering a secondary immune response [6]. This mechanism could explain the simultaneous presence of anti-GFAP and anti-MOG antibodies.

Subsequent PE resulted in marked improvement of these residual neurological symptoms, ultimately enabling independent ambulation. This stepwise therapeutic response suggests that corticosteroids were effective in controlling systemic inflammatory activity, whereas PE played a critical role in removing circulating pathogenic factors contributing to persistent neurological dysfunction. PE is thought to exert therapeutic effects not only through the removal of pathogenic autoantibodies but also by eliminating circulating pro-inflammatory cytokines and immune complexes that sustain CNS inflammation [10]. Therefore, in overlapping autoimmune CNS syndromes, escalation to PE should be considered when neurological recovery remains incomplete after corticosteroid therapy.

Furthermore, long-term management requires careful monitoring. While GFAP astrocytopathy may follow a monophasic course, the presence of MOG antibodies increases the likelihood of a relapsing-remitting course [4, 5]. Therefore, maintaining moderate-dose oral prednisolone after discharge was deemed necessary to prevent recurrence [4].

### Conclusion

This case illustrates that overlapping autoimmune CNS disorders should be suspected when clinical manifestations, such as severe encephalopathy or hyponatremia, cannot be fully explained by a single antibody. Early identification of anti-MOG antibodies and anti-GFAP antibodies and prompt initiation of PE may be considered in achieving favorable outcomes in steroid-refractory cases.

## Data Availability

The data supporting the findings of this study are available from the corresponding author upon reasonable request.

## Abbreviations

ADC: apparent diffusion coefficient
ADEM: acute disseminated encephalomyelitis
AMPH: amphiphysin
ANA: antinuclear antibody
AQP4: aquaporin-4
APS: antiphospholipid syndrome
CASPR2: contactin-associated protein-like 2
CL-β2GP1: cardiolipin–β2 glycoprotein I
CNS: central nervous system
CSF: cerebrospinal fluid
CV2 (CRMP5): collapsin response mediator protein 5
dsDNA: double-stranded DNA
DWI: diffusion-weighted imaging
FLAIR: fluid-attenuated inversion recovery
GAD65: glutamic acid decarboxylase 65
GFAP: glial fibrillary acidic protein
HIV: human immunodeficiency virus
IgG: immunoglobulin G
IgLON5: immunoglobulin-like cell adhesion molecule LON5
IVMP: intravenous methylprednisolone pulse
LGI1: leucine-rich glioma-inactivated 1
MOG: myelin oligodendrocyte glycoprotein
MOGAD: myelin oligodendrocyte glycoprotein antibody–associated disease
MRI: magnetic resonance imaging
MRA: magnetic resonance angiography
NMDA: N-methyl-D-aspartate
PE: plasma exchange
PNMA2 (Ma2): paraneoplastic Ma antigen 2
Recoverin: recoverin protein
Ri (ANNA-2): Ri antigen
SOX1: SRY-box transcription factor 1
SSA: Sjogren’s syndrome–related antigen A
SSB: Sjogren’s syndrome–related antigen B
Titin: titin protein
TPO: thyroid peroxidase
Tr (DNER): delta/notch-like epidermal growth factor–related receptor
Yo (PCA-1): Yo antigen
Zic4: zinc finger protein of the cerebellum 4
